# Supplementary material for: Prolonged Supplementation of Ozonated Sunflower Oil Bestows an Antiaging Effect, Improves Blood Lipid Profile and Spinal Deformities, and Protects Vital Organs of Zebrafish (Danio rerio) against Age-Related Degeneration: Two-Years Consumption Study
Source: Antioxidants (Basel). 2024 Jan 19;13(1):123. doi: 10.3390/antiox13010123 (PMC10812828; doi:10.3390/antiox13010123)
Supplement: Supplementary file 1 [file antioxidants-13-00123-s001.zip › antioxidants-2760466-Supplementary video-highlight.pptx]

## Slide 1
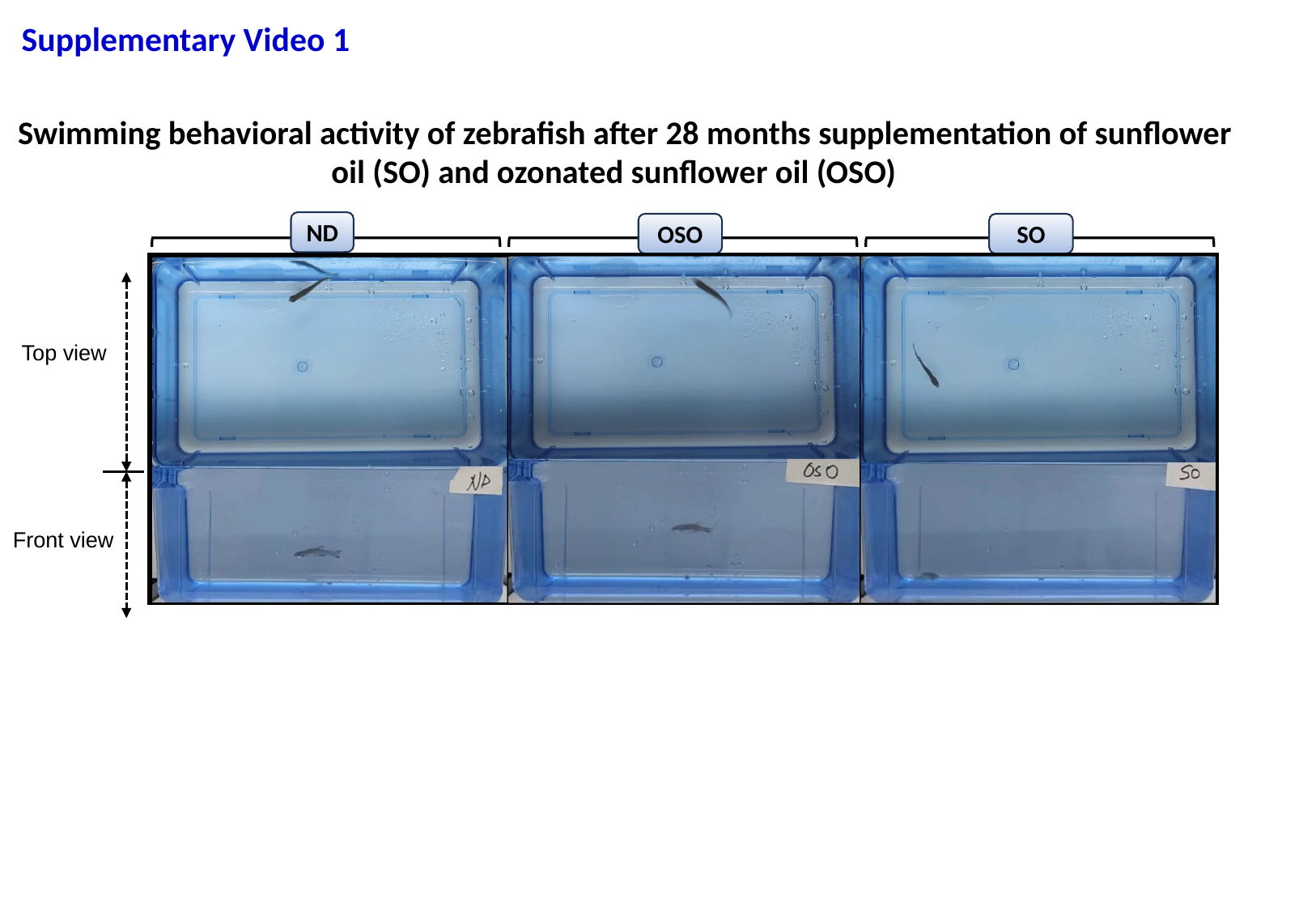

Supplementary Video 1
Swimming behavioral activity of zebrafish after 28 months supplementation of sunflower oil (SO) and ozonated sunflower oil (OSO)
ND
OSO
SO
Top view
Front view
