# Supplementary material for: Prolonged Supplementation of Ozonated Sunflower Oil Bestows an Antiaging Effect, Improves Blood Lipid Profile and Spinal Deformities, and Protects Vital Organs of Zebrafish (Danio rerio) against Age-Related Degeneration: Two-Years Consumption Study
Source: Antioxidants (Basel). 2024 Jan 19;13(1):123. doi: 10.3390/antiox13010123 (PMC10812828; doi:10.3390/antiox13010123)
Supplement: Supplementary file 1 [file antioxidants-13-00123-s001.zip › antioxidants-2760466-supplementary.pdf]

Supplementary material

Supplementary method M1: For the extraction, the SO or OSO mixed ND (1 g) was suspended in 2.5 mL of ethanol and agitated vigorously, following 30 min shaking (at 120 rpm). The ethanol phase was collected, and the remaining sample was resuspended in 2.5 mL of ethanol and shaken at 120 rpm for another 30 minutes. The ethanol phase was again recovered and combined with the previously extracted ethanol phase. The presence of SO and OSO was detected by comparing the UV-visible (185-400 nm) spectra with the standard SO and OSO spectra (prepared by suspending SO or OSO in ethanol). The amount of SO or OSO in the ND was determined by comparing the SO or OSO recovered from ND (using ethanol extraction). The standard curve of SO ($y=0.7325x+0.3255$) or OSO ($y=0.5431x+0.3421$) (obtained from the 0.25-2 mg/mL concentration range) was employed to quantify SO and OSO. The standard solutions of varied concentrations of SO and OSO were prepared by dissolving SO and OSO in absolute ethanol.

Supplementary Figure S1.

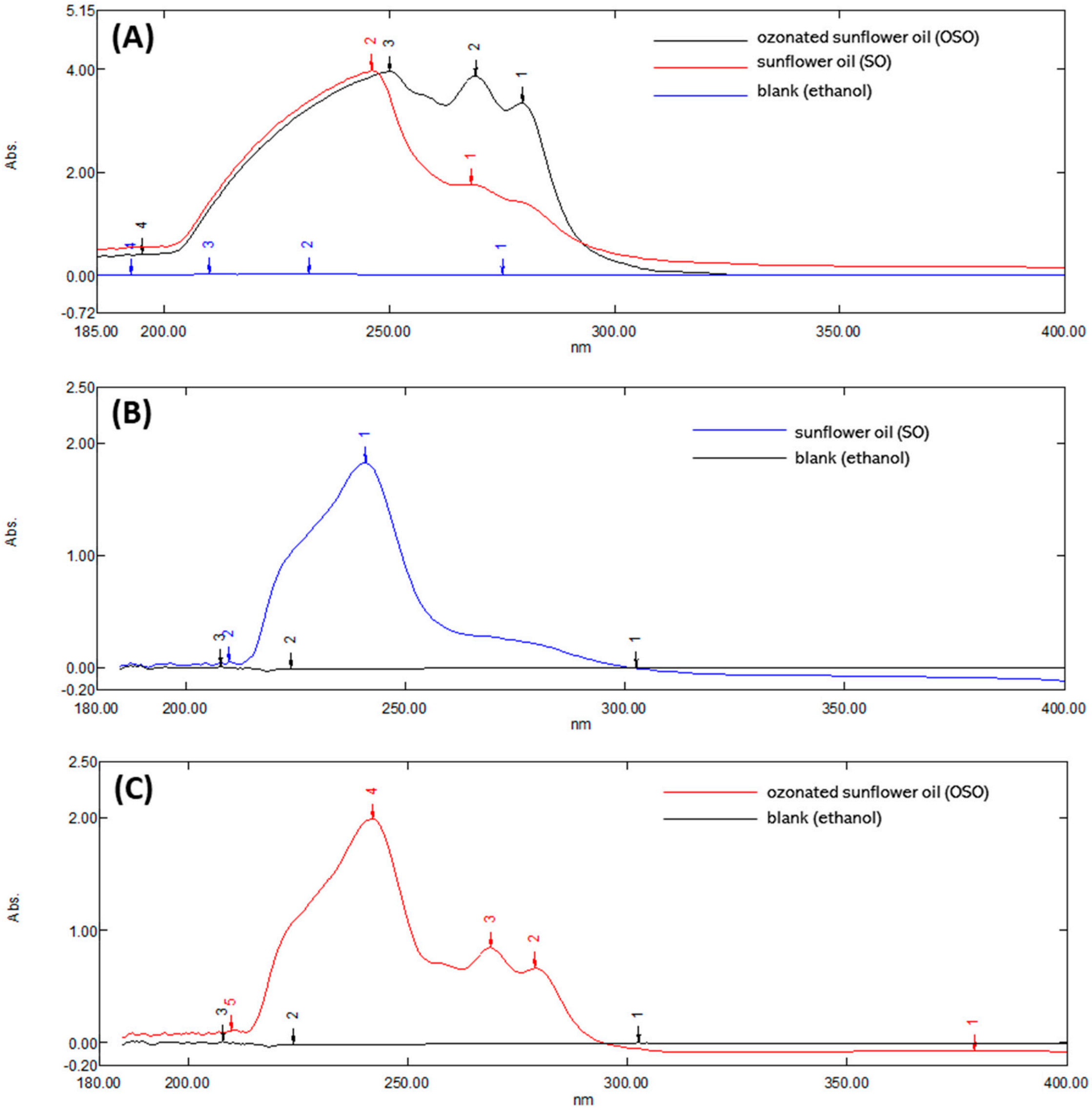

Supplementary Figure S1. UV spectrum (185 nm-400 nm) of **(A)** standard sunflower oil (SO) and ozonated sunflower oil (OSO). **(B)** Sunflower oil (SO) extracted from the ND. **(C)** Ozonated sunflower (OSO) extracted from the ND.

Supplementary Figure S2.

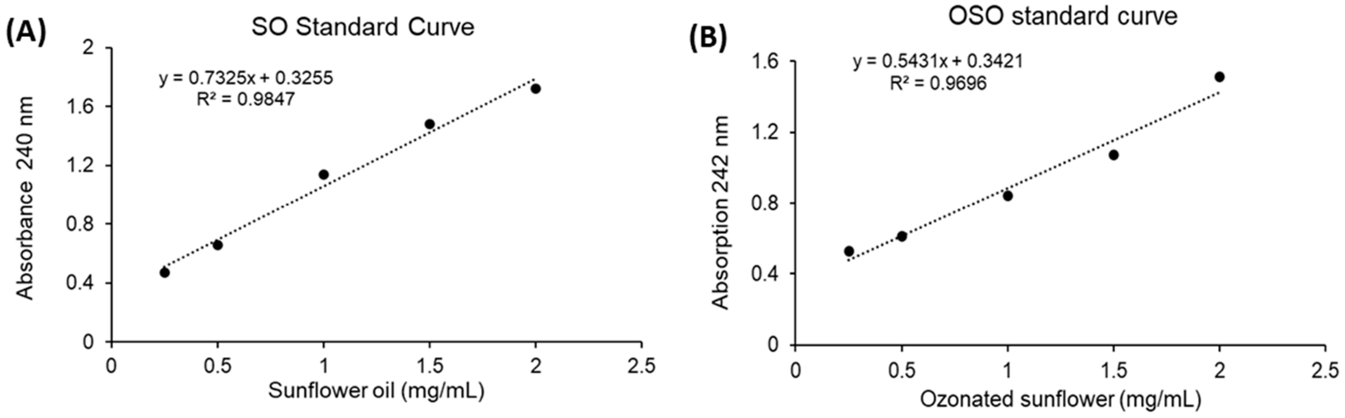

Supplementary Figure S2. Standard curve of **(A)** Sunflower oil (SO) and **(B)** Ozonated sunflower (OSO) at the concentration range 0.5 mg/mL to 2 mg/mL.

Supplementary Figure S3.

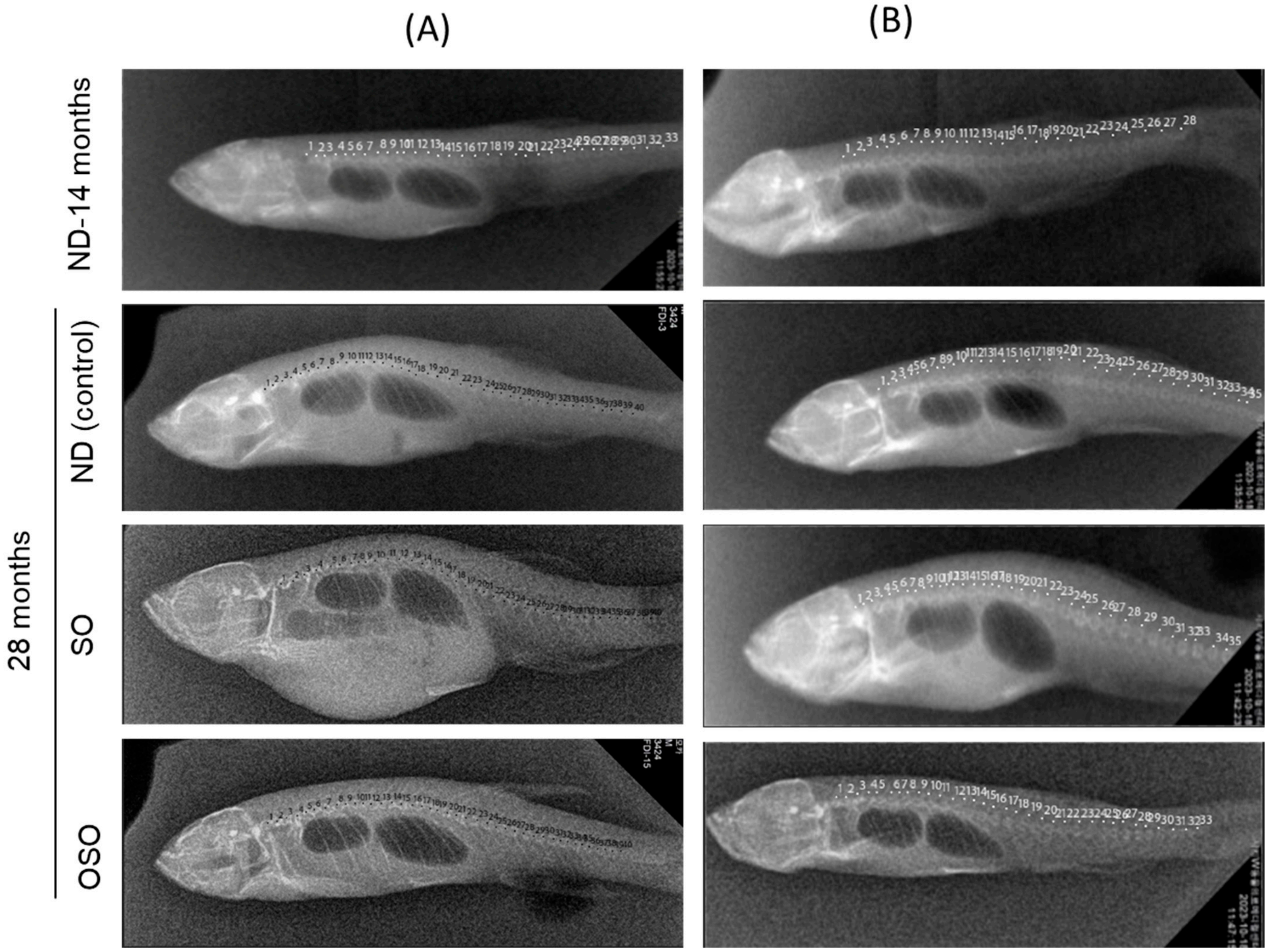

Supplementary Figure S3. X-ray images of zebrafish after 14 months supplementation of normal diet (ND) and 28 months supplementation of normal diet (ND), sunflower oil (SO) and ozonated sunflower oil (OSO).

Supplementary Figure S4.

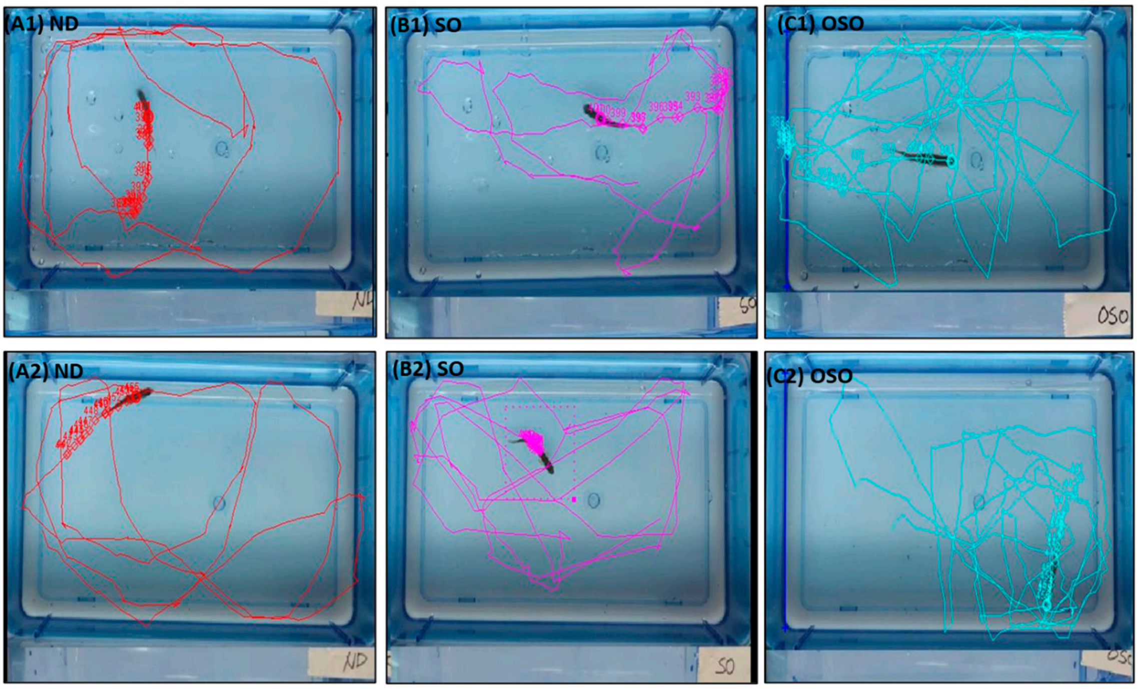

Supplementary Figure S4. Swimming trajectory of zebrafish at 28 months supplementation of **(A1-A2)** normal diet (ND), **(B1-B2)** normal diet + 20 % sunflower oil (SO, *wt/wt*) and **(C1-C2)** normal diet + 20% ozonated sunflower oil (OSO, *wt/wt*). of the zebrafish. Swimming trajectory of the zebrafish was determined by Tracker video analysis and modelling tool (available at https://physlets.org/tracker accessed on 16 May 2023).

Supplementary Figure S5.

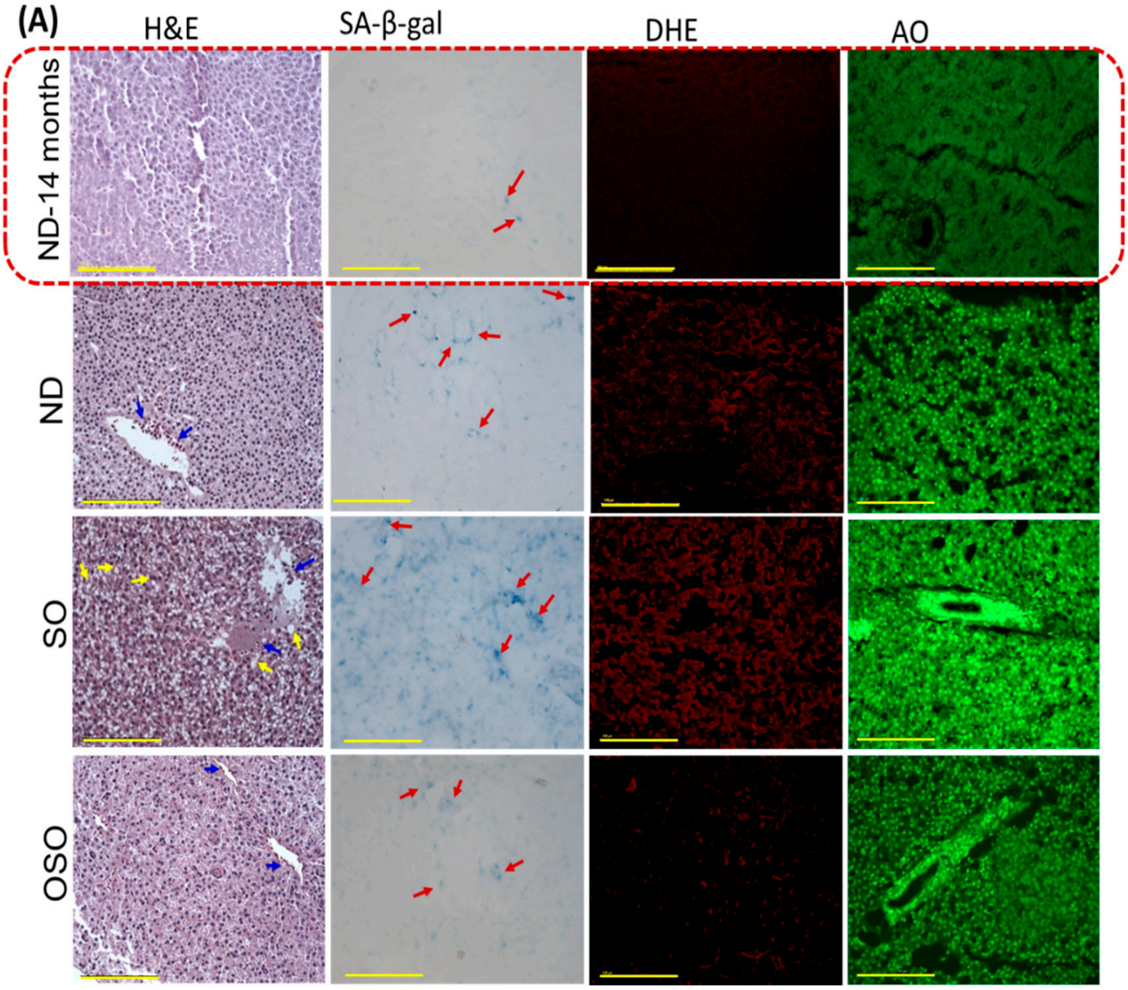

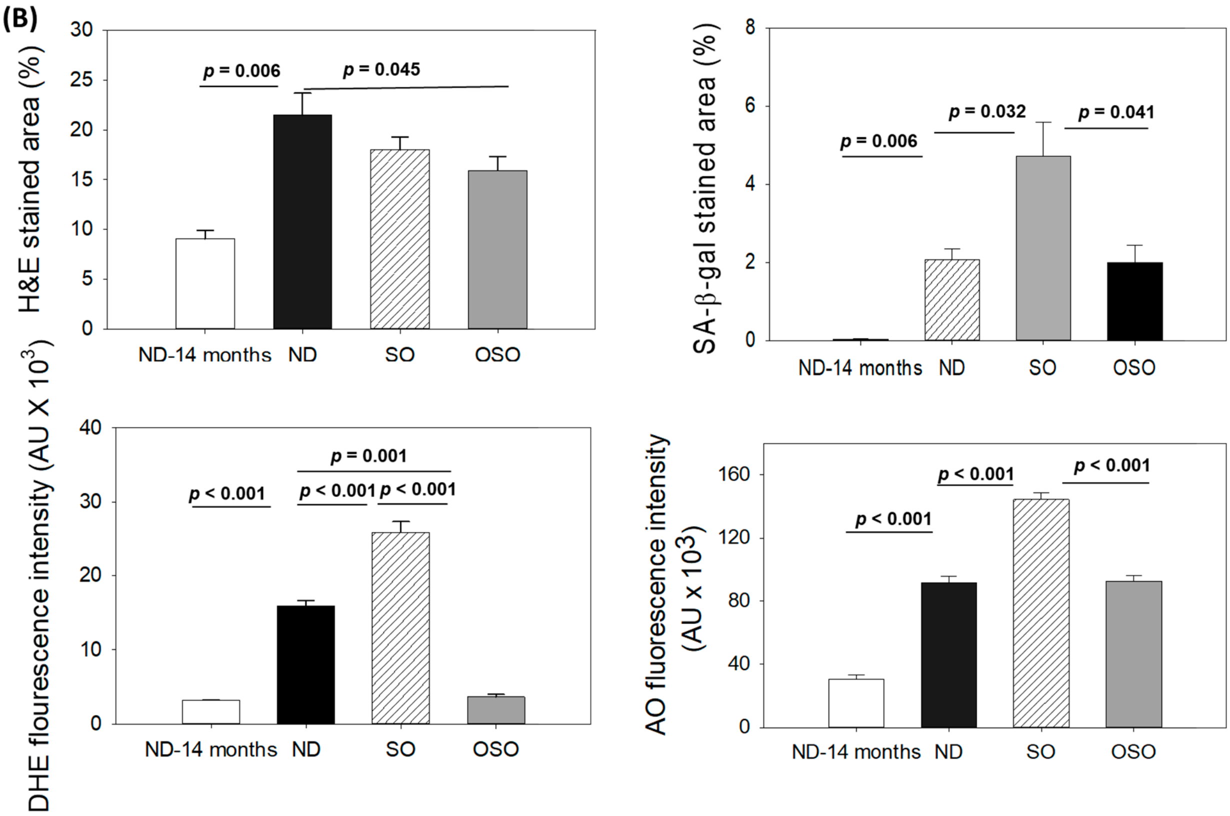

Supplementary Figure S5. A comparative assessment of 14 months of control (normal diet) and 24 months of supplementation of sunflower oil (SO) and ozonated sunflower oil (OSO) on the hepatic tissue of zebrafish. **(A)** Hematoxylin and eosin (H&E) staining, senescence-associated β galactosidase (SA-β-gal) staining, dihydroethidium (DHE) and acridine orange (AO) fluorescent staining. [0.1 mm, scale bar]. **(B)** Image j-based quantification of H&E and SA-β-gal-stained area, fluorescent intensities of DHE and AO-stained area. ND-14 months represents the control normal diet feed zebrafish at 14 months; ND represents the control normal diet; SO represents ND supplemented with 20% SO (*wt/wt*); OSO represents ND supplemented with 20% OSO (*wt/wt*). The p value signifies the statistical significance discerned between groups resulting from the one-way ANOVA following Tukey's Post Hoc analysis.

Supplementary Figure S6.

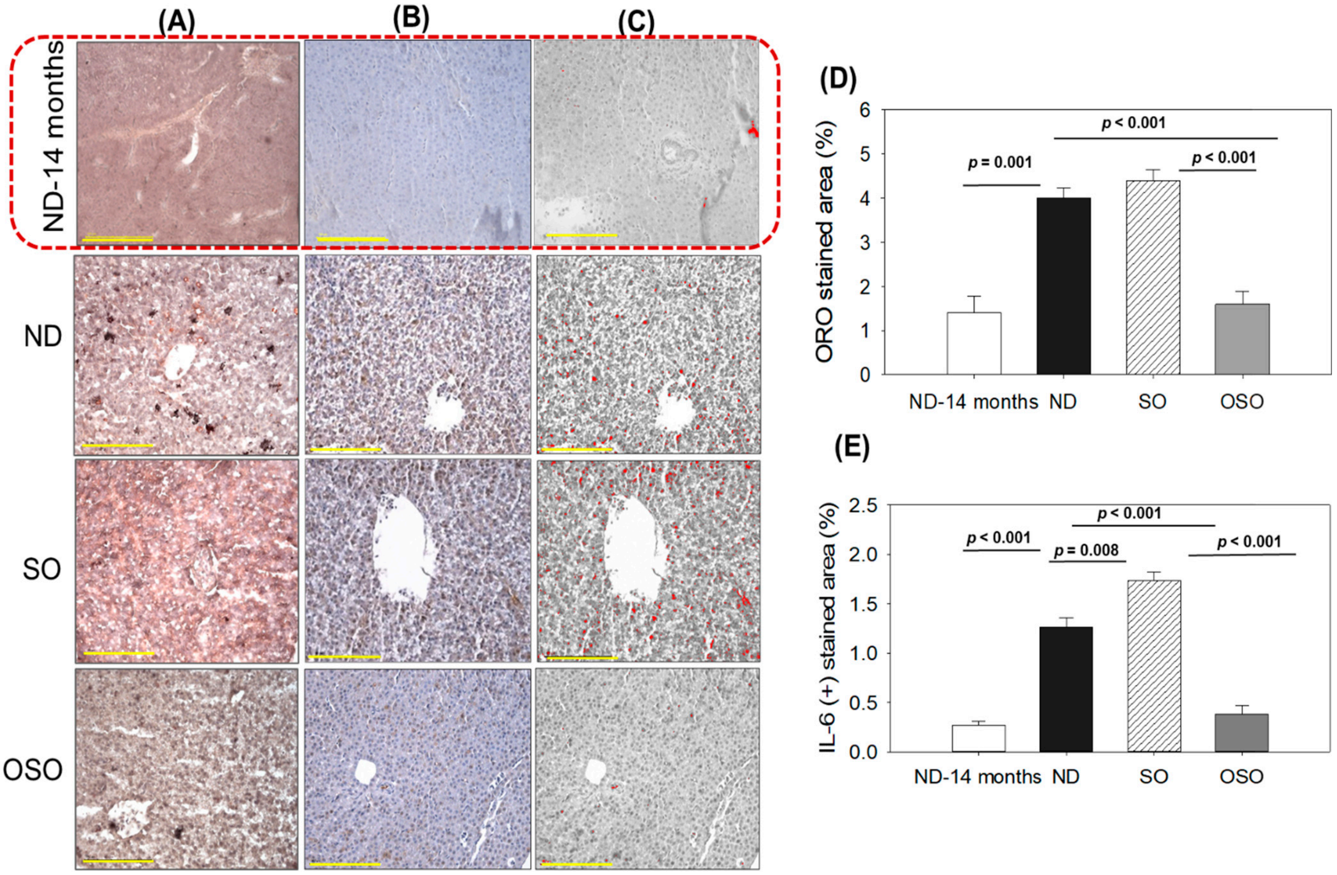

Supplementary Figure S6. A comparative assessment of 14 months of control (normal diet) and 24 months of supplementation of sunflower oil (SO) and ozonated sunflower oil (OSO) on fatty liver alterations and IL-6 production in zebrafish. **(A)** Oil red O staining. **(B)** Immunohistochemistry (IHC) for the assessment of IL-6 generation [0.1 mm, scale bar]. **(C)** Employing Image J software (http://rsb.info.nih.gov/ij/ assessed on 2023 Jan 30), the brown color of the native IL-6-stained area has interchanged with red color, employing a brown color threshold value from 20 to 120. to intensify the visualization of IHC stained area. **(D)** and **(E)** Quantification oil red O stained and IL-6-stained area employing image J software. ND-14 months represents the control normal diet feed zebrafish at 14 months; ND represents the control normal diet; SO represents ND supplemented with 20% SO (*wt/wt*); OSO represents ND supplemented with 20% OSO (*wt/wt*). The p value signifies the statistical significance discerned between groups resulting from the one-way ANOVA following Tukey's Post Hoc analysis.
